# Heart in Disguise: Unmasking a Novel Gene Deletion in Dilated Cardiomyopathy

**DOI:** 10.7759/cureus.55170

**Published:** 2024-02-28

**Authors:** Moyan Sun, Vikas Kilaru, Hussain Majeed, Sharvil Patel, Aleksandros Mihilli, Giancarlo Acosta

**Affiliations:** 1 Internal Medicine, Northeast Georgia Medical Center Gainesville, Gainesville, USA; 2 Cardiology, Northeast Georgia Medical Center Gainesville, Gainesville, USA

**Keywords:** dystrophinopathy, guideline-directed medical therapy (gdmt), nonischemic cardiomyopathy, rare genetic disorder, heart failure, duchenne muscular dystrophy (dmd), dilated cardiomyopathy (dcm)

## Abstract

Dilated cardiomyopathy (DCM) is an underrecognized condition with a myriad of etiologies, but it is often labeled idiopathic. However, genetic mutations are emerging as a more common cause of idiopathic DCM than previously believed. Herein, we present a case of a previously healthy 45-year-old woman who presented with three weeks of exertional dyspnea and orthopnea. An echocardiogram showed DCM with severely reduced systolic function and diastolic dysfunction.

She was extensively worked up for potential etiologies of her heart failure which included HIV testing, parasite smear, viral serologies, autoimmune testing, cardiac MRI for infiltrative diseases, and coronary catheterization. She was ultimately tested for genetic mutations which revealed a 49-51 exon deletion of the dystrophin (Duchenne muscular dystrophy (DMD)) gene.

This case highlights the guideline-based evaluation and management of new-onset heart failure in a healthy 45-year-old female without known predisposing risk factors or family history. It also sheds light on the expansive genetic etiologies that have only recently been identified in those with idiopathic cardiomyopathy. Further research is crucial to improve our understanding of genetic associations of cardiomyopathy.

## Introduction

Dystrophinopathies encompass a spectrum of X-linked disorders characterized by pathogenic variants in the dystrophin (Duchenne muscular dystrophy (DMD)) gene. The DMD gene encodes for dystrophin, a protein responsible for cross-linking muscle cytoskeleton with the extracellular matrix. Intragenic deletions of the DMD gene account for approximately 65% of dystrophinopathies, and several specific mutations have been previously identified [1]. The most recognized pathology is DMD which often manifests as severe, early-onset systemic muscular weakness. Followed by Becker muscular dystrophy, which is less common and typically presents with a later onset and milder clinical course [2]. All dystrophinopathies are X-linked and therefore almost always affect males. However, the recognition of DMD-associated dilated cardiomyopathy (DCM), particularly in middle-aged females with no skeletal muscle involvement, is a noteworthy departure from previous convention [3]. Research has shown that deletion of 48-49 exons may lead to an earlier onset of cardiomyopathy with worse outcomes. Conversely, deletion of 51-52 exons may play a cardioprotective role [4]. However, a deletion of exons 49-51, as in this case, has been undescribed until now.

## Case presentation

A 45-year-old previously healthy Caucasian woman presented to the emergency department (ED) with three weeks of progressive dyspnea and orthopnea. She also reported paroxysmal nocturnal dyspnea, nausea, vomiting, and 15-pound unintentional weight loss. She had never used tobacco, alcohol, or illicit drugs. She had no significant medical history and took no medications. She was adopted, but she has four healthy children. Initial vitals showed blood pressure of 107/87 mmHg, heart rate of 95 beats/min, respiratory rate of 18 breaths/min, and temperature of 98.5F. Physical examination was notable for jugular venous pressure of 16 cm H_2_O, bibasilar crackles, normal cardiac sounds without murmurs, and 1+ bilateral lower extremity edema. The initial laboratory results obtained in the ED are delineated in Table 1.

**Table 1:** Laboratory results NT-pro-BNP: N-terminal pro-brain natriuretic peptide

Labs	Value	Reference range
Sodium	142	135-148 mmol/L
Potassium	3.8	3.5-5.2 mmol/L
Blood urea nitrogen	26	5-23 mg/dL
Glucose	100	65-99 mg/dL
Creatinine	1.07	0.8-1.3 mg/dL
White blood cells	8.5	4.8-10.8 K/uL
Hemoglobin	13.5	14-18 g/dL
Platelets	350	130-400 K/uL
NT-pro-BNP	6,305	< 900 pg/dL
High-sensitivity troponin I	0.03	< 0.04 ng/mL
Erythrocyte sedimentation rate	7.0	0-20 mm
C-reactive protein	6.0	0.00-0.60 mg/dL
Thyroid-stimulating hormone	2.573	0.34-4.82 mIU/mL

Her electrocardiogram (ECG) showed normal sinus rhythm and left-bundle branch block. Computed tomography (CT) of the chest showed bibasilar ground glass opacities but no pulmonary embolus. An echocardiogram was obtained, which revealed a left ventricular ejection fraction (LVEF) of 15% with severe dilation (LVIDd 6.1cm), grade III diastolic dysfunction, and severe mitral regurgitation (Videos 1-2).

**Video 1:** Parasternal long-axis view from initial transthoracic echocardiogram showing reduced left ventricular ejection fraction and reduced right ventricular function

**Video 2:** Apical four-chamber view from initial transthoracic echocardiogram showing reduced left ventricular ejection fraction and reduced right ventricular function

She was extensively worked up for potential causes of her DCM. Viral etiologies were investigated with sputum culture and polymerase chain reaction (PCR) but were negative. HIV testing was negative. Parasitic causes were investigated with serum immunoglobulins but were negative. Autoimmune, Quantiferon testing, and blood and sputum bacterial cultures were also negative.

Intravenous diuresis with furosemide was initiated, leading to gradual clinical improvement of her dyspnea and orthopnea. Attempts at initiating guideline-directed medical therapy (GDMT) were limited by brief episodes of hypotension. At this point, the underlying etiology remained unclear, and cardiac ischemic testing was pursued. Coronary catheterization showed normal coronary arteries and right atrial pressure of 16 mmHg, pulmonary capillary wedge pressure of 28 mmHg, cardiac index of 1.5 l/min/m^2^, and pulmonary vascular resistance of 3.016 WU.

She was eventually started on metoprolol 25 mg daily, spironolactone 25 mg daily, and dapagliflozin 10 mg daily. To assess cardiac function improvement and evaluate alternative etiologies, contrast cardiac magnetic resonance imaging and stress imaging revealed persistent severe dilation of LV with LVEF of 12% but no evidence of prior infarction, fibrosis, or myocarditis. She was discharged with close follow-up in the heart failure clinic where a molecular genetic test (massively parallel sequencing (MPS)) showed that she was heterozygous for the exons 49-51 deletion in the DMD gene.

Furthermore, she exhibited no symptoms and maintained euvolemia. Nevertheless, her blood pressure continued to hover at the borderline low range, hindering the initiation of an angiotensin receptor-neprilysin inhibitor or angiotensin-converting enzyme inhibitor. Iron deficiency anemia was found, and she is scheduled for iron sucrose infusions. Ongoing close follow-ups are planned, with a gradual up-titration of GDMT. Additionally, a three-month surveillance echocardiogram has been scheduled to evaluate left ventricular recovery.

## Discussion

DCM is characterized by enlarged ventricles with poor contractility. The etiologies of DCM can be classified by ischemic (59%) and non-ischemic causes (41%), such as stress, infections, autoimmune diseases, and nutrition deficiencies [5]. Often, non-ischemic DCM is labeled as idiopathic as no etiology can be readily identified. However, with more sensitive modalities of testing such as cardiac MRI, many previously thought idiopathic cases of DCM are now thought to be secondary to genetic mutations [6].

DCM is the most common form of cardiomyopathy and is typically idiopathic or familial. Familial DCM is an inherited single-gene disorder that typically follows an autosomal dominant pattern with variable expressivity and reduced penetrance. Mitochondrial mutations demonstrating a maternal pattern of inheritance also contribute. While autosomal recessive mutations are less prevalent, X-linked recessive inheritance is associated with many different genetic mutations including emerin, dystrophin, and tafazzin [7]. At least 50 different single genes have been identified as contributing to familial DCM. Genetic testing uses multi-gene panels to screen for these genes simultaneously. Some of these genes encode essential structural proteins involved in the nuclear membrane, sarcomere, and Z band. Research into the broad range of involved genes can help begin explaining the variable presentations of more severe and early-onset forms of DCM [7].

Similarly to idiopathic DCM, genetic testing has led to the elucidation of specific phenotypes within previously thought classical dystrophin mutation phenotypes leading to dystrophinopathies. The most recognized dystrophinopathy is DMD, which often manifests as severe, early-onset systemic muscular weakness, followed by Becker muscular dystrophy, which is typically later onset with a milder clinical course [8]. All dystrophinopathies are X-linked and, therefore, almost always affect males. However, the recognition of DMD-associated DCM, particularly in middle-aged females with no skeletal muscle involvement, is a noteworthy departure from previous conventions [3,4].

Presently, consensus guidelines advocate for genetic testing in individuals diagnosed with primary cardiomyopathy. This approach has led to an enhanced detection of pathogenic gene mutations [8,9]. Moreover, the consequences of identifying distinct phenotypic manifestations extend beyond improving the recognition of those at risk, contributing to more robust data for formulating medical therapy guidelines. As these cases are being identified, it has led to a better understanding of the polymorphic presentation that can occur. In a recent cross-sectional study of 53 asymptomatic women with pathogenic variants of the DMD gene, 66% were found to have structural, functional, and conduction abnormalities [8]. Due to the innate variability of various mutations within the spectrum of DMD, there has been some speculation that a specific exon study could provide insight into the cardiac dysfunction experienced in terms of clinical manifestations and time to onset. For example, earlier development of cardiomyopathy could be related to mutations in exons 48-49, as seen in this case, while mutations in exons 51-52 could lead to a reduction in myocardial dysfunction and cardioprotective properties [9,10]. Unfortunately, a deletion at exons 49-51 - which could involve both mechanisms - has been undescribed previously; there are no current treatments specifically for this mutation. Current literature suggests treating patients according to current heart failure guidelines. If ventricular dysfunction persists upon follow-up, patients would be evaluated for appropriateness for heart transplantation [9,10]. As a result, identifying at-risk patients with early detection of cardiac involvement is paramount to the initiation of intervention prior to the onset of symptoms [10].

## Conclusions

This case highlights the intricate interplay between clinical presentation, diagnostic challenges, and the ultimate genetic revelation in a complex case of DCM. The 49-51 exons deletion of the DMD gene provides a reminder that genetic factors can influence cardiac health. As we advance into an era of patient-centered medicine, incorporating genetic testing into routine clinical practice holds the promise of unraveling the complexities of cardiomyopathies. More research is needed to determine the extent of association between specific genetic mutations and DCM.

## References

[1] Muntoni F, Torelli S, Ferlini A (2003). Dystrophin and mutations: one gene, several proteins, multiple phenotypes. Lancet Neurol.

[2] Ulm EA, Nagaraj CB, Tian C, Smolarek TA (2023). Identification of Biallelic dystrophin gene variants during maternal carrier testing for Becker muscular dystrophy and review of the DMD exon 49-51 deletion phenotype. Mol Genet Genomic Med.

[3] Morales A, Mahajan K (2023). Dystrophinopathies. StatPearls.

[4] Perez-Sanchez I, Sabater-Molina M, Rocamora ME, Glover G, Escudero F, de Mingo Casado P, Gimeno-Blanes JR (2018). Phenotypic characterization of a family with an in-frame deletion in the DMD gene and variable penetrance. Curr Gene Ther.

[5] Manolio TA, Baughman KL, Rodeheffer R (1992). Prevalence and etiology of idiopathic dilated cardiomyopathy (summary of a National Heart, Lung, and Blood Institute Workshop). Am J Cardiol.

[6] Burkett EL, Hershberger RE (2005). Clinical and genetic issues in familial dilated cardiomyopathy. J Am Coll Cardiol.

[7] McNally EM, Golbus JR, Puckelwartz MJ (2013). Genetic mutations and mechanisms in dilated cardiomyopathy. J Clin Invest.

[8] Solheim TÅ, Fornander F, Raja AA (2021). Cardiac involvement in women with pathogenic dystrophin gene variants. Front Neurol.

[9] Shore S, Grau-Sepulveda MV, Bhatt DL (2015). Characteristics, treatments, and outcomes of hospitalized heart failure patients stratified by etiologies of cardiomyopathy. JACC Heart Fail.

[10] Hershberger RE, Givertz MM, Ho CY (2018). Genetic evaluation of cardiomyopathy-a Heart Failure Society of America practice guideline. J Card Fail.

